# Dramatic Decrease in Prevalence of Soil-Transmitted Helminths and New Insights Into Intestinal Protozoa in Children Living in the Chaco Region, Bolivia

**DOI:** 10.4269/ajtmh.14-0039

**Published:** 2015-04-01

**Authors:** Fabio Macchioni, Higinio Segundo, Simona Gabrielli, Valentina Totino, Patricia Rojas Gonzales, Esteban Salazar, Ricardo Bozo, Alessandro Bartoloni, Gabriella Cancrini

**Affiliations:** Dipartimento di Scienze Veterinarie, Università di Pisa, Pisa, Italy; Distrito de Salud Cordillera, Departamento de Santa Cruz, Camiri, Plurinational State of Bolivia; Dipartimento di Sanità Pubblica e Malattie Infettive, Università “Sapienza”, Roma, Italy; Dipartimento di Medicina Sperimentale e Clinica, Università di Firenze, Firenze, Italy

## Abstract

We assessed the prevalence of intestinal parasites among 268 2–12-year-old children living in rural areas, small villages, and semi-urban areas of the Chaco region, south-eastern Bolivia. The overall parasitism was 69%. Only protozoa, helminths, or co-infections were observed in 89.2%, 5.9%, or 4.9% of the positive children, respectively. A significant progressive increase in overall parasite prevalence was found when passing from rural areas to small villages and semi-urban areas. The most commonly found species were *Entamoeba coli* (38.4%), *Giardia intestinalis* (37.7%), and *Blastocystis* spp. (16%). *Hymenolepis nana* was the most prevalent helminth (5.6%), followed by *Ascaris lumbricoides* and hookworms (1.5% and 0.4%) evidenced only in rural areas and in villages. Molecular diagnostics identified *Blastocystis* subtypes 9 and 2, and 5 infections by *Entamoeba histolytica* and 4 by *Entamoeba dispar*. The dramatic decrease in prevalence of soil-transmitted helminths with respect to that observed about 20 years ago (> 40%) evidences the success of the preventive chemotherapy intervention implemented in 1986. Health education and improved sanitation should be intensified to control protozoan infections.

In developing countries the lack of access to safe water, sanitation, and hygiene are the key factors for the high prevalence of intestinal protozoa that, in infants and children, frequently have the clinical expression of malabsorption syndrome and gastrointestinal morbidity.^1^ Moreover, *Ascaris lumbricoides* may contribute to nutritional deficiencies and even produce intestinal occlusion, whereas other soil-transmitted helminths (STHs) cause chronic intestinal blood loss that results in anemia, and impairing physical growth, cognition, learning and working capacities.^2^

In the Santa Cruz Department (Plurinational State of Bolivia), studies conducted ∼40 years ago showed intestinal parasitism ranging from 85.4% to 99.5%, with 65% of polyparasitism.^3^–^5^ A survey carried out in 1987 detected an overall value of 79%, with prevalence of STHs up to 64%.^6^,^7^ Further investigations conducted in 1990 in children living in two rural communities showed prevalence of STH infections of 41% and 64%, respectively.^8^ Starting in 1986, the Bolivian Ministry of Health developed a Parasitic Disease Control Program based on preventive chemotherapy with mebendazole that is still part of the Integral Attention to Prevalent Childhood Diseases Program (AIEPI).

The study reported herein, programmed and carried out in 2011 with the support of the Guaraní political organization (Asamblea del Pueblo Guaraní) and in agreement with the Bolivian Ministry of Health (who gave the Ethical approval), was aimed at evaluating the current prevalence of intestinal parasites in apparently healthy children. Results of the analyses were reported daily, and positive subjects had immediate access to further specific medical check-up and drug treatment.

A total of 268 randomly selected children (120 boys and 148 girls) 2–12 years of age were enrolled. Sample collection was performed in rural communities (Añimbo, Arenal, Brecha, Mandiyuti, Taputá, Timboirenda, Uruguay), small villages (San Antonio del Parapetí, Espino, Ivicuati), and semi-urban areas (Boyuibe, La Brecha, Cuevo, Lagunillas), ~3 months after the last delivering of preventive chemotherapy. Stool specimens daily collected, in the afternoon were submitted to microscopic examination, in a drop of iodine solution, of both wet smears and sediments after Ridley concentration.^9^ Parasites were identified on the basis of their morphological features.^10^ Samples positive for *Entamoeba histolytica* complex and for *Blastocystis* spp. were further analyzed by polymerase chain reaction (PCR) amplification and sequencing to identify species/subtypes involved. Genomic DNA was extracted using the NucleoSpin tissue kit (Macherey-Nagel, Düren, Germany) and a 540-bp (and in the nested PCR a 374-bp) fragment of the 30-kDa surface antigen from *E. histolytica* complex and the small subunit rRNA gene of *Blastocystis* spp. were PCR amplified following published protocols.^11^,^12^ Amplicons were purified (SureClean kit, Aurogene, Rome, Italy) and sequenced (Eurofins MWG Operon, Ebersberg, Germany). Sequences obtained were corrected, aligned, and compared with *Blastocystis* SSU rRNA gene sequences available database (http://www.ncbi.nlm.nih.gov). Subtypes (STs) were identified according to the classification proposed by Stensvold and others in 2007.^13^

Statistical analysis (chi-square [χ^2^] or Fisher's exact tests) was applied to the results, and *P* values < 0.05 were considered significant.

Intestinal protozoa and/or eggs from helminths were recovered in 185 of 268 children (69.0%). Only protozoa were evidenced in 165 subjects (89.2%). Helminths, and protozoa associated to helminths, were detected in 5.9% and 4.9% of the positive children, respectively. The most commonly found parasite was *Entamoeba coli*, followed by *Giardia intestinalis*, and *Blastocystis* spp. (Table 1). An overall 54.6% of the positive subjects harbored two or more species, and *G. intestinalis* was the protozoan more often found in single infections (43.6%). As for helminths, *Hymenolepis nana* was the species more frequently identified (overall prevalence: 5.6%), whereas eggs of *A. lumbricoides* and hookworms were rarely present (their prevalence was 1.5% and 0.4%), always in co-infection with protozoa, and eggs of other Cestoda (*Taenia* spp.) were occasionally found.

No differences in prevalence of parasitism were evidenced by sex (44.9% versus 44.3%) or by age. Lower parasitism rates (48.4% and 50%, respectively) were found in the youngest and oldest subjects, whereas 4- to 6-year-old children had the highest rate (77.6%). The analysis of the results by area (Table 2) showed significant differences (*P* = 0.01) as a result of the progressive increase of overall parasite prevalence when passing from rural areas to small villages and semi-urban areas. This trend is mainly caused by *E. coli*, *G. intestinalis*, and *H. nana*, suggesting that indirect/direct contamination with human feces (even fresh), or unidentified zoonotic reservoirs is easier in these more crowded settings with inadequate sanitation. On the contrary, STHs were found, at very low prevalence, only in rural areas and in villages.

Sequencing analysis applied to *Blastocystis*-microscopically single positive showed high identity (98–100%) to *Blastocystis* ST9 (*N* = 8) and ST2 (*N* = 4) deposited in GenBank (accession nos. AM275366.1 and AM275376.1).^13^,^14^ The ST2 is a very commonly found subtype, whereas ST9 has so far only been isolated from humans and on very few occasions. Finally, nested PCR identified five infections caused by *E. histolytica* and four to *E. dispar*.

Our study showed a high intestinal parasitism rate (69%) in children living in the Bolivian Chaco. However, it was significantly lower than that observed in 1987, in the adult and children population living in the same area (79%) (*P* = 0.005).^7^ Poor general hygienic and sanitary conditions may explain the maintained high presence of *E. coli* (38%) and *G. intestinalis* (38%), not significantly different from that observed in 1987 (41%, *P* = 0.6 and 31%, *P* = 0.06, respectively).^6^ Waterborne transmission and close contact with animals (not changed during these years) could play an important role in the transmission of both these species and the two zoonotic STs of *Blastocystis,* whose pathogenicity is still under debate.^15^,^16^

Our findings evidenced a low prevalence of the *E. histolytica* complex (≈5%), in general agreement with that reported in the Santa Cruz region (0.4–10%),^17^ but lower than that reported in the valleys and in the Northern Bolivian Altiplano (0–38.6%).^18^ However, using molecular diagnostics we were able to discriminate a high proportion of invasive *E. histolytica* (5 of 9) from the morphologically identical *Entamoeba dispar* (4 of 9), thus directing important therapeutic decisions.

The most relevant finding is the dramatic decrease in prevalence of STHs with respect to that observed about 20 years ago (hookworm from up to 50% to 0.4%, *A. lumbricoides* from up to 19% to 1.5%, *Trichuris trichiura* from up to 19 to 0%).^8^ These findings contrast with prevalence for hookworm (23%), *A. lumbricoides* (29%), and *T. trichiura* (32%) in a school-aged population of the Cordillera province, estimated in a recent study based on a geostatistical model.^19^ Furthermore, although not statistically significant, we observed a decrease in prevalence of *H. nana* (6% versus 9%, *P* = 0.14), with respect to that observed in 1987.^6^ Evidence of *Taenia* eggs supports data on the serological reactive response to *Taenia solium* antigens shown by adult subjects living in this area and corroborates the role of cysticercosis as a health problem for the investigated areas.^20^

Although our study was not aimed at analyzing factors influencing epidemiological trends, we consider that implementation of the control program (delivery of single dose mebendazole to the 2- to 9-year-old child population administered approximately every 6 months), started in 1986, led to the expected results. Indeed, up-to-date knowledge of local STH prevalence, as provided in this study, should help advance public health policies that need to balance the detrimental influence of these parasites on child health with the economic and ecologic costs of continued mass distribution chemotherapeutic prevention strategies if they are no longer needed. Although our data set is relatively small and further studies may be necessary before interrupting regular deworming programs in the study area, the very low prevalence of STHs in the surveyed children is within the World Health Organization (WHO)-recommended range to reduce the frequency of drug administration (to every 2 years) and monitor the possible recrudescence of the infections.^21^

In conclusion, our findings confirm preventive chemotherapy as a valid measure to reduce the prevalence of soil-transmitted helminths,^21^ but also the need for continuing the efforts in control strategies, including health education and improving access to sanitation.

## Figures and Tables

**Table 1:** Intestinal parasites found (in single and multiplex infection) in 268 children living in different communities of the Chaco region (Bolivia)

Species	Prevalence *N* (%)	Relative prevalence (%)	Single parasitism *N* (%)	Co-parasitism *N* (%)
*Giardia intestinalis*	101 (37.7)	54.6	44 (43.6)	57 (56.4)
*Chilomastix mesnili*	3 (1.1)	1.6	1 (33.3)	2 (66.7)
*Entamoeba coli*	103 (38.4)	55.7	17 (16.5)	86 (83.5)
*Endolimax nana*	16 (6.0)	8.6	2 (12.5)	14 (87.5)
*Iodamoeba butschlii*	10 (3.7)	5.4	0	10 (100)
*E. histolytica* complex	9 (3.4)	4.9	1 (11.1)	8 (88.9)
*E. hartmanni*	3 (1.1)	1.6	0	3 (100)
*Blastocystis* spp.	43 (16.0)	23.2	11 (25.6)	32 (74.4)
*Hymenolepis nana*	15 (5.6)	8.1	8 (53.3)	7 (46.7)
*Taenia* spp.	3 (1.1)	1.6	0	3 (100)
*Ascaris lumbricoides*	4 (1.5)	2.2	0	4 (100)
Hookworms	1 (0.4)	0.5	0	1 (100)
Total	185 (69.0)		84 (45.4)	101 (54.6)

**Table 2:** Parasites more frequently detected in children living in the Chaco region (Bolivia), by environment

Species	Rural areas	Small villages	Semi-urban areas
	Positive/examined *N* (%)		
Total	34/58 (58.6)	28/44 (63.6)	123/165 (74.5)*
*G. intestinalis*	15/58 (25.9)	13/44 (29.5)	73/165 (44.2)*
*E. coli*	14/58 (24.1)	16/44 (36.4)	74/165 (44.8)*
*Blastocystis* spp.	11/58 (19.0)	8/44 (18.2)	24/165 (14.5)
STHs	3/58 (5.2)	1/44 (2.3)	0/165 (0)
*H. nana*	1/58 (1.7)	2/28 (7.1)	12/165 (7.3)

* Statistically significant differences.
